# Brucellosis emergence in the Canadian Arctic

**DOI:** 10.1016/j.onehlt.2024.100712

**Published:** 2024-03-21

**Authors:** Xavier Fernandez Aguilar, Fabien Mavrot, Om Surujballi, Lisa-Marie Leclerc, Kugluktuk Angoniatit Association, Ekaluktutiak Hunters and Trappers Organization, Olokhaktomiut Hunters and Trappers Committee, Matilde Tomaselli, Susan Kutz

**Affiliations:** a Faculty of Veterinary Medicine, University of Calgary, Calgary, AB T2N 4Z6, Canada; b Canadian Food Inspection Agency, Ottawa Laboratory Fallowfield, 3851 Fallowfield Road, PO Box 11300, Station H, Nepean, ON K2H 8P9, Canada; c Department of Environment, Government of Nunavut, P.O. Box 377, Kugluktuk, NU X0B 0E0, Canada; d Kugluktuk Angoniatit Association, 13 Klengenberg St., Kugluktuk, NU X0B 0E0, Canada; e Ekaluktutiak Hunters & Trappers Organization, 4 Mitik St. Cambridge Bay, NU X0B 0C0, Canada; f Olokhaktomiut Hunters & Trappers Committee, P.O. Box 161, Ulukhaktok, NT X0E 0S0, Canada; g Polar Knowledge Canada, Canadian High Arctic Research Station, 1 Uvajuq Road, PO Box 2150, Cambridge Bay, Nunavut X0B 0C0, Canada

**Keywords:** *Brucella suis* biovar 4, Zoonoses, Caribou, Muskox, Wildlife health surveillance, Emerging diseases

## Abstract

Brucellosis is an important zoonotic disease affecting animals and subsistence harvesters in the circumarctic. We investigated recent trends (2015–2022) of brucellosis seropositivity in caribou (*Rangifer tarandus*) and muskoxen (*Ovibos moschatus*) in the Central Canadian Arctic by using data from community-based wildlife health surveillance programs. The overall sample prevalence of *Brucell*a antibodies was 10.0% (*n* = 271) in muskoxen and 15.5% (*n* = 277) in caribou. Sample seroprevalence in muskoxen varied geographically with an increasing trend of exposure on NW Victoria Island (from 0% to 36.8% between 2016 and 2022; Kendall tau = 0.283, *p* = 0.001). The presence of *Brucella suis* biovar 4 was confirmed by culture from clinical cases in this area. Our results indicate that *Brucella suis* biovar 4 continues to circulate in the Central Canadian Arctic in caribou and muskoxen and may be now circulating in muskoxen independently from caribou. These findings highlight the need to better understand the ecology and drivers of brucellosis emergence in Arctic multi-host systems.

## Highlights

• Brucellosis is a threat to wildlife and people from the Central Canadian Arctic.
• Community-based health surveillance allowed investigations between 2015 and 2022.
• Overall seroprevalence was 10.0% in muskoxen and 15.5% in sympatric caribou.
• A local emergence up to 36.8% is documented in muskoxen from NW Victoria Island.
• *Brucella suis* biovar 4 may be circulating in muskoxen independently from caribou.

## Graphical abstract

**Figure:**
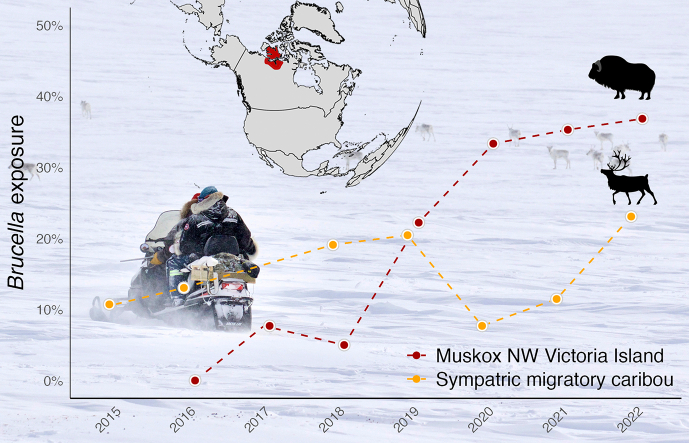

Brucellosis has been recognized in the Arctic since the 20th century. *Brucella suis* biovar 4 (BS4) is the main causative agent for brucellosis in Arctic and subarctic regions, in both animals and people [1]. It is commonly referred as rangiferine brucellosis because the primary hosts are caribou and reindeer (*Rangifer tarandus*), yet it also infects other sympatric wildlife like muskoxen (*Ovibos moschatus*) [2] or moose (*Alces alces*) [3]. People are exposed to BS4 while butchering or consuming infected wildlife or through the husbandry and food products from domestic reindeer [1]. The presence of brucellosis in wildlife poses a significant health risk to the Inuit and other Indigenous people, which is particularly concerning as subsistence hunts are a cornerstone of their cultural heritage and are an important component of Arctic food security [4].

In the Canadian Arctic during the 1980s–90s, brucellosis was reported in barren-ground caribou herds from the mainland [5,6], as well as in caribou from Baffin Island [7]. Local knowledge from the Kitikmeot region, Nunavut, indicated that brucellosis was a recent problem in caribou at that time [5]. During the same decades, cases of brucellosis in Indigenous people were regularly diagnosed in the same regions [[7], [8], [9]]. More recent serological surveys between 2007 and 2014, indicated no exposure or very low exposure to *Brucella* in mainland barren-ground caribou herds (0% to 5%), suggesting a different scenario than the previously described [10].

In contrast, a reversed trend in *Brucella* exposure have been recorded in the Dolphin and Union (DU) caribou herd, in which neither antibodies nor clinical signs of the disease were detected between 1986 and 1990 (*n* = 62) [5], but antibodies against *Brucella* were detected in 16.3% of the caribou sampled between 2015 and 2021 [11]. This herd is a unique Designatable Unit as assigned by the Committee on the Status of Endangered Wildlife in Canada (COSEWIC) [12], and performs a seasonal migration between its winter range on the mainland Nunavut and its summer range on Victoria Island where they calve, in the Arctic Archipelago (Fig. 1).

Changes of brucellosis in Victoria Island have also been recorded in muskoxen. Tomaselli et al. 2019, provided compelling evidence that brucellosis was present in muskoxen on Victoria island and had increased from a very low prevalence (0.9%) before 2001 to up to 5.6% by 2016 in the vicinity of the community of Cambridge Bay [2]. That study compiled data from participatory epidemiology, post-mortem examinations, serology from commercial harvests, and confirmed bacteriological cases; however, different diagnostic tests and sample types were used in that study, and trends were difficult to interpret.

In response to an apparent recent increase in submissions of abnormal findings compatible with brucellosis (e.g. abscesses, swollen joints, swollen testicles, etc.) in muskoxen and caribou between 2016 and 2022, we initiated a study to assess recent trends of *Brucella* infection in caribou and muskoxen from Victoria Island and the adjacent mainland. These data are part of a community-based wildlife health surveillance network established in the region through the collaborative partnerships among three Indigenous communities from the Northwest Territories (Ulukhaktok) and Nunavut (Kugluktuk and Cambridge Bay), territorial governments (Government of Nunavut (GN) and Government of Northwest Territories (GNWT)), the federal government (Polar Knowledge Canada) and the Kutz Research Group (Faculty of Veterinary Medicine, University of Calgary) [11]. Samples obtained per year varied depending on communities as sampling schemes were affected by local funding and the COVID-19 pandemic.

To assess *Brucella* antibodies, blood on Nobuto filter paper strips (Toyo Roshi Kaisha, Ltd., Tokyo, Japan) were collected by Inuit hunters from muskoxen and caribou harvested for food, and by wildlife biologists from caribou captures [13]. Inuit harvesters also collected abnormal tissues detected while butchering the animals and submitted them to the Kutz Research Group and/or Alberta Centre of the Canadian Wildlife Health Cooperative (CWHC) for a pathological diagnosis. Caribou samples were tested for *Brucella* antibodies with a competitive ELISA [14] and muskox samples with an indirect ELISA [15] at the National Brucellosis Reference Laboratory (Canadian Food Inspection Agency - Ottawa Animal Health Laboratory). Both assays were modified to test fluid eluted from Nobuto filter strips instead of serum [14]. Because *Brucella* antibodies are rarely detected in young animals, we only used data from animals older than 2 years old to assess temporal trends [1]. Sample size per year is shown in the supplementary table. We investigated trends in serological status (positive/negative) over time using a permutation test approach, which allows for the assessment of the significance of observed trends while accounting for potential biases introduced by non-random sampling. Specifically, the Kendall tau statistic was calculated based on the observed data, and 1000 permutations were performed to generate a null distribution of the test statistic. The *p*-value was calculated as the proportion of permuted test statistics that were as extreme as or more extreme than the observed test statistic. We analyzed all caribou data together, as all individuals are part of the same migratory herd. For muskoxen, we conducted separate analyses by area, as this species does not undertake extensive seasonal migrations, and populations on islands and the mainland are treated as distinct (refer to the Table 1 and Fig. 1 for area descriptions).

The overall sample seroprevalence in muskoxen was 10.0% (*n* = 271, 95% CI: 6.9–14.1) and in caribou from the DU herd was 15.5% (*n* = 277, 95% CI: 11.7–20.3). For muskoxen, seropositive cases were restricted to those from Victoria Island and the mainland east of Bathurst Inlet (area E), and prevalence varied by area between 5.3% (area C, near Cambridge Bay) and 21.3% (area A, near Ulukhaktok) (Table 1, Fig. 1). The observed Kendall tau statistic for the trend in serological status over time in muskoxen from NW Victoria Island (area A, near Ulukhaktok) was 0.283, indicating a moderate positive association between time and serological status. The permutation test revealed a significant *p*-value of 0.001, suggesting that the observed trend in serological status over time is statistically significant after accounting for potential biases. No significant trends were detected in muskoxen from other geographical areas. During the years of the study, the presence of BS4 in muskoxen was confirmed by culture isolation from clinical cases in muskoxen, which were also detected at an increasing frequency in NW Victoria Island (area A, near Ulukhaktok; 1 in 2018, 1 in 2019, 2 in 2021; 5 in 2022; Goldsmith et al. unpublished data). No clear trend was detected in seropositive caribou (Table 1), yet BS4 was isolated from seven harvested caribou between 2018 and 2022 [11].

Brucellosis was confirmed for the first time by BS4 isolation in a muskox on NW Victoria Island in 1996, but no seropositive muskoxen were detected around this time in the area, 0% (*n* = 405, 1994–1999) [2]. Our findings provide new and further evidence that brucellosis is emerging in muskoxen, with a recent significant increase on NW Victoria Island since 2016 (area A, near Ulukhaktok). In this area, the sample prevalence in muskoxen is the highest ever reported in this species, and it is higher than in the seasonally sympatric DU caribou herd (Table 1). Furthermore, according to most recent surveys and local knowledge from Inuit communities, caribou density on NW Victoria Island is low (see [16,17] and Fig. 1). It is generally assumed that *Rangifer tarandus* (caribou and reindeer) serve as the primary hosts for BS4. Therefore, these species are also believed to be the ones maintaining BS4-brucellosis foci in the Arctic. Previous reports of brucellosis in muskoxen were sporadic and typically occurred in areas sympatric with infected caribou herds [5,18]. Our results indicate an emerging and enzootic BS4 infection in muskoxen from NW Victoria Island suggesting that the ecology of BS4 may have changed and deserves further attention. In contrast, the sample prevalence in muskoxen in SE Victoria Island (area C), around Cambridge Bay, may have declined in recent years. However, muskox numbers in this area have drastically declined and very few animals are currently harvested, which complicates the interpretation of the results. Of additional concern is the increasingly regular detection of seropositive and clinically affected muskoxen (Kutz et al., unpubl. data) on the mainland on Kent Peninsula and around Ellice River (Fig. 1, Area E).

**Table 1:** Sample prevalence of *Brucella* antibodies in muskoxen by geographic area and Dolphin and Union caribou, older than 2 years old, from the Kitikmeot and the Inuvialuit regions of the Central Canadian Arctic. Victoria Island (SE: around Cambridge Bay; NW: around Ulukhaktok; SW: Pin-3/Franklin point area), and Mainland (E: East of Bathurst Inlet, including Kent Peninsula; W: West Bathurst Inlet, around Kugluktuk). Confidence intervals (95%) are shown in brackets.

Herd/Area	n	2015	2016	2017	2018	2019	2020	2021	2022	Total
**Muskoxen**										
Victoria Island NW (Area A)	94	NA	0.0 (0.0–49.0)	7.7 (0.4–33.3)	5.0 (0.3–23.6)	22.2 (9.0–45.2)	33.3 (1.7–79.2)	35.3 (17.3–58.7)	36.8 (19.1–59-0)	21.3 (14.2–30.6)
Victoria Island SW (Area B)	13	NA	NA	10.0 (0.5–40.4)	0.0 (0.0–56.1)	NA	NA	NA	NA	7.7 (0.4–33.3)
Victoria Island SE(Area C)	38	NA	10.0(0.5–40.4)	11.1(0.6–43.5)	0.0(0.0–43.4)	0.0(0.0–65.8)	0.0(0.0–25.9)	0.0(0.0–94.9)	NA	5.3(1.4–17.3)
Mainland W(Area D)	94	NA	NA	0.0(0.0–29.9)	0.0(0.0–8.0)	0.0(0.0–8.6)	NA	NA	NA	0.0(0.0–3.9)
Mainland E (Area E)	32	NA	0.0 (0.0–94.9)	0.0 (0.0–56.1)	14.3 (5.0–34.6)	0.0 (0.0–56.1)	50.0 (2.6–97.4)	0.0 (0.0–65.8)	NA	12.5 (5.0–28.1)
**Caribou**										
Dolphin and Union herd⁎	277	10.7 (3.7–27.2)	13.0 (4.5–32.1)	0 (0–39.0)	19.1 (12.3–28.5)	20.4 (11.1–34.5)	7.7 (0.4–33.3)	11.5 (5.7–21.8)	23.1 (8.2–50.3)	15.5 (11.7–20.3)

n: sample size; NA: samples were not collected or not analyzed yet.

⁎ Part of these results have previously been published [10,11].

**Fig. 1:**
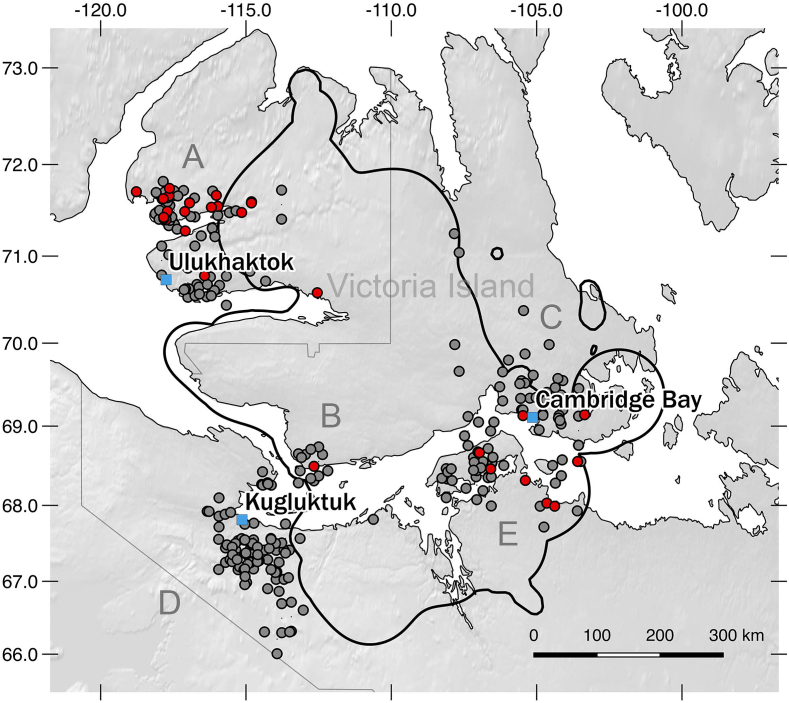
Spatial distribution of seropositivity for *Brucella* in hunted muskoxen from the Inuvialuit (Northwest Territories, left to the grey line including Ulukhaktok) and Kitikmeot (Nunavut, right to the grey line including Kugluktuk and Cambridge Bay) regions of the central Canadian Arctic, 2016–2021. Red dots indicate positive results and grey dots negative results. The blue squares are the locations of the communities. The thick black line depicts the Dolphin and Union caribou annual range using a kernel analysis of telemetry data from adult cows collected between 1997 and 2006 and 2015–2020 (Government of Nunavut). The geographic areas within the study region are indicated in grey letters; A: NW Victoria Island; B: SW Victoria Island; C: SE Victoria Island; D: Mainland West of Bathurst Inlet; E: Mainland East of Bathurst Inlet including Kent Peninsula.

The high sample seroprevalence (21.3%) of brucellosis in muskoxen on NW Victoria Island, together with its persistence on the mainland East of Bathurst Inlet, suggest that this bacterium may now be circulating in muskoxen independently of caribou in some areas, and as such, it may continue expanding its geographic range. Genetic analyses of strains found in muskoxen and caribou will further elucidate transmission dynamics between these species [19].

Our findings of apparent emergence of brucellosis in muskoxen and apparently persistent prevalence in the Dolphin and Union caribou herd, together with a recent report documenting increased prevalence in caribou from Alaska [20], underscores that this pathogen remains an important threat for wildlife and people in the Arctic. Long-term fluctuations in brucellosis have been reported in infected caribou herds [1]. However, changes in infection dynamics appear to occur gradually over the course of years, even decades, with complete clearance of the infection not conclusively documented [1]. Brucellosis foci in wildlife pose an exposure risk to people that rely on wildlife for food, and our results warn that this risk may be increasing. Brucellosis in caribou is fairly well known by harvesters and public health officials alike, yet the disease syndrome in muskoxen is relatively new and unexpected in some areas, thus posing a potential elevated risk. Efforts to enhance wildlife health surveillance and incorporate public health risk management for brucellosis and other zoonotic diseases in the Arctic are essential. Equally important is the application of One Health approaches that encompass not only the scientific aspects but also the Indigenous Knowledge and the cultural, social, and ecological dimensions of wildlife health in Indigenous peoples from the Arctic [21,22]. While sampling is opportunistic and sample sizes are limited, our collaborative community-based wildlife health surveillance networks have already provided a mechanism to detect, monitor and respond to emerging disease threats [23]. Continued efforts to maintain and improve such networks, together with further research, are needed to understand the ecology and drivers of emerging diseases in the Arctic.

## Funding

This work was supported by grants to S.K. from 10.13039/100012258Polar Knowledge Canada (project NST-1718-0015), ArcticNet, Shikar Foundation and Canada North Outfitting (1052116), 10.13039/501100008638Environment and Climate Change Canada (GXE20C347), Irving Maritime Shipbuilding (project 1041735), and NSERC; a grant from the Aboriginal Fund for Species at Risk to the Olokhaktomiut Hunters and Trappers Committee (GCXE21C168); a Nunavut General Monitoring Program grant to the Ekaluktutiak Hunters and Trappers Organization (NGMP project #19EC66); and a 10.13039/100001250Morris Animal Foundation fellowship (D20ZO-407) to X.F.A.

## CRediT authorship contribution statement

**Xavier Fernandez Aguilar:** Conceptualization, Data curation, Formal analysis, Funding acquisition, Investigation, Visualization, Writing – original draft. **Fabien Mavrot:** Investigation, Methodology, Project administration, Writing – review & editing. **Om Surujballi:** Investigation, Methodology, Validation, Writing – review & editing. **Lisa-Marie Leclerc:** Funding acquisition, Methodology, Project administration, Writing – review & editing. **Matilde Tomaselli:** Funding acquisition, Project administration, Resources, Writing – review & editing. **Susan Kutz:** Conceptualization, Funding acquisition, Investigation, Project administration, Resources, Supervision, Validation, Writing – review & editing.

## Declaration of competing interest

The authors declare that they have no known competing financial interests or personal relationships that could have appeared to influence the work reported in this paper.

## Data Availability

Data will be made available on request.

## Appendix A Supplementary data

Supplementary table with the number of blood samples analyzed for the detection of *Brucella* antibodies by study area and year of collection. Image 1
