# Supplementary material for: Brucellosis emergence in the Canadian Arctic
Source: One Health. 2024 Mar 21;18:100712. doi: 10.1016/j.onehlt.2024.100712 (PMC11000101; doi:10.1016/j.onehlt.2024.100712)
Supplement: Supplementary file 1 — Supplementary table with the number of blood samples analyzed for the detection of Brucella antibodies by study area and year of collection. [file mmc1.pdf]

One Health, ELSEVIER

Supplementary Materials - Brucellosis emergence in the Canadian Arctic

Xavier Fernandez Aguilar, Fabien Mavrot, Om Surujballi, Lisa-Marie Leclerc, Kugluktuk Angoniatit Association, Ekaluktutiak Hunters & Trappers Organization, Olokhaktomiut Hunters & Trappers Committee, Matilde Tomaselli, Susan Kutz

Supplementary table. Number of samples analyzed by study area and year of collection for the detection of *Brucella* antibodies in blood from caribou and muskoxen, >2 years old, from the Kitikmeot and the Inuvialuit regions of the Central Canadian Arctic. Victoria Island (SE: around Cambridge Bay; NW: around Ulukhaktok; SW: Pin-3/Franklin point area), and Mainland (E: East of Bathurst Inlet, including Kent Peninsula; W: West Bathurst Inlet).

Herd/Area	2015	2016	2017	2018	2019	2020	2021	2022	Total
**Muskoxen**									
Victoria Island SE (Area C)	NA	10	9	5	2	11	1	NA	38
Victoria Island NW (Area A)	NA	4	13	20	18	3	17	19	94
Victoria Island SW (Area B)	NA	NA	10	3	NA	NA	NA	NA	13
Mainland E (Area E)	NA	1	3	21	3	2	2	NA	32
Mainland W (Area D)	NA	NA	9	44	41	NA	NA	NA	94
**Caribou**									
Dolphin and Union herd	28	23	6	89	44	13	61	13	277

NA: samples were not collected or not analyzed yet.
